# Identification of hepatic NPC1L1 as an NAFLD risk factor evidenced by ezetimibe‐mediated steatosis prevention and recovery

**DOI:** 10.1096/fba.2018-00044

**Published:** 2019-02-13

**Authors:** Yu Toyoda, Tappei Takada, Masakazu Umezawa, Fumiya Tomura, Yoshihide Yamanashi, Ken Takeda, Hiroshi Suzuki

**Affiliations:** ^1^ Department of Pharmacy The University of Tokyo Hospital Tokyo Japan; ^2^ Research Institute for Science and Technology Organization for Research Advancement, Tokyo University of Science Chiba Japan; ^3^ Present address: Faculty of Pharmaceutical Sciences Sanyo‐Onoda City University Yamaguchi Japan

**Keywords:** cholesterol re‐uptake, drug repositioning, lipid homeostasis, NAFLD model animal, toll‐like receptor, transporter

## Abstract

Non‐alcoholic fatty liver disease (NAFLD) is a serious global public health concern. Nevertheless, there are no specific medications for treating the associated abnormal accumulation of hepatic lipids such as cholesterol and triglycerides. While seminal findings suggest a link between hepatic cholesterol accumulation and NAFLD progression, the molecular bases of these associations are not well understood. Here, we experimentally demonstrate that hepatic Niemann‐Pick C1‐Like 1 (NPC1L1), a cholesterol re‐absorber from bile to the liver, can cause steatosis, an early stage of NAFLD using genetically engineered L1‐Tg mice characterized by hepatic expression of NPC1L1 under the control of ApoE promoter. Contrary to wild‐type mice that have little expression of hepatic Npc1l1, the livers of L1‐Tg mice fed a high‐fat diet became steatotic within only a few weeks. Moreover, hepatic NPC1L1‐mediated steatosis was not only prevented, but completely rescued, by orally administered ezetimibe, a well‐used lipid‐lowering drug on the global market, even under high‐fat diet feedings. These results indicate that hepatic NPC1L1 is an NAFLD‐exacerbating factor amendable to therapeutic intervention and would extend our understanding of the vital role of cholesterol uptake from bile in the development of NAFLD. Furthermore, administration of a TLR4 inhibitor also prevented the hepatic NPC1L1‐mediated steatosis formation, suggesting a latent link between physiological roles of hepatic NPC1L1 and regulation of innate immune system. Our results revealed that hepatic NPC1L1 is a novel NAFLD risk factor contributing to steatosis formation that is rescued by ezetimibe; additionally, our findings uncover feasible opportunities for repositioning drugs to treat NAFLD in the near future.

**Abbreviations**
B.W.: body weight
CFD: control‐fat diet
Eze: ezetimibe
GO: gene‐ontology
HFD: high‐fat diet
L/B ratio: the ratio of liver weight to body weight
L1‐Av: NPC1L1‐expressing adenovirus
L1‐Tg mice: transgenic mice expressing human NPC1L1 in hepatocytes
NAFLD: non‐alcoholic fatty liver disease
NASH: non‐alcoholic steatohepatitis
NPC1L1: Niemann‐Pick C1‐Like 1
TG: triglyceride
TLR4: toll‐like receptor 4
WT: wild‐type

## 1 INTRODUCTION

Non‐alcoholic fatty liver disease (NAFLD) is one of the most frequent hepatic disorders associated with a food‐abundant lifestyle.^1^ This global health problem represents a large spectrum of hepatic conditions ranging from steatosis (also known as fatty liver and characterized by the hepatic accumulation of lipids such as cholesterol and triglycerides [TG]) to non‐alcoholic steatohepatitis (NASH; a risk factor of liver fibrosis and cirrhosis) .^2^ Although steatosis, an early stage of NAFLD, is relatively benign form, its progression can induce further intrahepatic damages. The prevention and cure of steatosis is therefore important for maintaining human health. Nevertheless, effective pharmacological treatments for NAFLD have not been established.

Hepatic lipid accumulation is part of the aetiology of NAFLD, which has attracted widespread interest by numerous investigations. Recent findings suggest that excess accumulation of cholesterol in the liver is associated with NAFLD progression.^3^, ^4^, ^5^ However, the main molecular basis of NAFLD exacerbation remains largely unknown, especially during the initial process; that is progressing from a healthy liver to steatosis. In mammalians, hepatic cholesterol is delivered not only into the blood but also into bile. This led us to reason that enhancing the biliary secretion of hepatic cholesterol itself could reduce the risk of NAFLD and that cholesterol re‐absorption from bile could have the opposite effect. Importantly, the human liver can secrete cholesterol into bile, but can also re‐uptake the biliary‐secreted cholesterol. The latter process is mediated by the Niemann‐Pick C1‐Like 1 (NPC1L1) protein, a cholesterol transporter that is expressed on the bile canalicular membrane in humans.^6^, ^7^ Thus, we hypothesized that hepatic NPC1L1 could exacerbate NAFLD, including steatosis.

In addition to the liver, *NPC1L1* is also expressed in the human small intestine and plays a pivotal role as a physiologically important cholesterol transporter: however, rodents such as mice show little *Npc1l1 *expression in the liver.^8^ Thereby, common animal models of NAFLD should show a poor ability to re‐absorb biliary cholesterols compared with humans. Owing to this species difference between humans and rodents, the involvement of hepatic NPC1L1 in the development/progression of NAFLD has been overlooked in many animal studies. Indeed, little is known about the biological effects of re‐absorbed cholesterol from bile on the hepatic condition. Therefore, to investigate whether hepatic NPC1L1 could increase the risk for hepatic disorders is an important issue.

The physiological function of NPC1L1 is selectively inhibited by ezetimibe, a globally used lipid‐lowering drug.^9^ While large clinical studies have been conducted to evaluate the efficacy of ezetimibe in dyslipidemia therapy,^10^, ^11^ little information is available regarding the therapeutic effect of ezetimibe on human NAFLD. Although its potential efficacy has been proposed in some reports ,^4^, ^12^ most such speculations appear to be extended interpretations based on general moderation of NAFLD (especially NASH) observed in commonly used cholesterol‐lowering medications.^13^ More problematically, few studies have experimentally demonstrated an anti‐NAFLD effect of ezetimibe in terms of the hepatic NPC1L1 inhibition. In this context, it is important to examine whether hepatic NPC1L1 can exacerbate steatosis (an initial feature of NAFLD), and whether ezetimibe can attenuate NPC1L1‐dependent hepatic disorder.

In this study, we investigated the physiological impact of hepatic NPC1L1 on the risk of developing NAFLD using transgenic mice expressing human NPC1L1 in hepatocytes (L1‐Tg mice).^6^ Surprisingly, L1‐Tg mice fed a western diet exhibited steatosis within only a few weeks. This phenotype was prevented and rescued by ezetimibe. We therefore concluded that hepatic NPC1L1 is a novel exacerbating factor of steatosis amendable to therapeutic intervention. Moreover, L1‐Tg mice may serve as a good animal model for better understanding of the developmental mechanisms underlying NAFLD and exploring the new therapeutic targets. Indeed, by using this model, we showed that Toll‐like receptor 4 (TLR4) antagonist could potentially serve as a therapeutic agent for steatosis.

## 2 MATERIALS AND METHODS

### 2.1 Materials

Critical materials and resources used in this study were summarized in Table 1. All other chemicals used were commercially available and were of analytical grade.

**Table 1:** Key resources

Reagent or resource	Source	Identifier
Antibodies		
Rabbit polyclonal anti‐NPC1L1	Novus Biologicals	Cat# NB400‐128; RRID: AB_10000815
Rabbit polyclonal anti‐EGFP	Life technologies	Cat# A11122; RRID:AB_221569
Rabbit polyclonal anti‐α‐tubulin	Abcam	Cat# ab15246; RRID: AB_301787
Donkey anti‐rabbit IgG‐horseradish peroxidase (HRP)‐conjugate	GE Healthcare	Cat# NA934V; RRID:AB_772206
Virus strains		
EGFP‐expressing adenovirus	Toyoda et al^14^	N/A
NPC1L1‐EGFP‐expressing adenovirus	This paper	N/A
Chemicals		
Ezetimibe	Sequoia Research Products	Cat# SRP04000e; CAS: 163222‐33‐1
IAXO101	Innaxon	Cat# IAX‐600‐001‐M005; CAS: 1202388‐64‐4
Clophosome^®^‐ Clodronate Liposomes (neutral)	FormuMax Scientific	Cat# F70101C‐N
Control Liposomes for Clophosome^®^ (neutral)	FormuMax Scientific	Cat# F70101‐N
Critical commercial assays		
Cholesterol E‐test Wako Kit	Wako	Cat# 439‐17501
Triglyceride E‐test Wako Kit	Wako	Cat# 432‐40201
RNeasy Micro Kit	Qiagen	Cat# 74004
Deposited data		
Gene microarray dataset	This paper	GEO: GSE110285
Expression profiling by array: Human liver biopsy of different phases from control to NASH	Ahrens et al^15^	GEO: GSE48452
Experimental models: organisms/strains		
Mouse: C57BL/6 J	Japan SLC	C57BL/6JJmsSlc
Mouse: B6;D2‐Tg(APOE‐NPC1L1)20Lqyu/J	The Jackson Laboratory	JAX: 008408
Oligonucleotides		
A full list of Primers	This paper	See Table 2
Recombinant DNA		
The complete NPC1L1 cDNA	Yamanashi et al^7^	GenBank: AY437867
Others		
Control fat diet: *consisted of crude sources of protein (soybean waste, whitefish meal, and yeast), fat (cereal germ and soybean oil), and carbohydrate (wheat flour and corn)*	CLEA Japan	Cat# CE‐2: http://www.clea-japan.com/en/diets/diet_a/a_03.html (Detail of ingredients)
High‐fat diet	CLEA Japan	Cat# D15002
ViraPower^TM^ Adenovial Gateway^TM^ Expression Kit	Invitrogen	Cat# K493000
Adeno‐X^TM^ Rapid Titer Kit	Clontech	Cat# 632250

### 2.2 Animals

All animals received humane care according to criteria outlined in the Guide for the Care and Use of Laboratory Animals prepared by the National Academy of Sciences and published by the National Institutes of Health. All animal experiments were performed according to methods approved by the Institutional Animal Care and Use Committee of the University of Tokyo.

L1‐Tg mice (B6;D2‐Tg(APOE‐NPC1L1)20Lqyu/J)^6^ were purchased from The Jackson Laboratory (Bar Harbor, Maine, USA) and backcrossed for at least eight generations to C57BL/6 J mice (Japan SLC, Inc, Shizuoka, Japan) before use. Hemizygous positive L1‐Tg mice were always crossed to wild‐type (WT) C57BL/6 J mice to generate hemizygous L1‐Tg mice and WT littermate controls for all experiments. PCR‐based genotyping for the L1‐Tg mice using their genomic DNA isolated from ear punch biopsy with hot NaOH was performed according to the Jackson Laboratory's instructions. The following primer sets were used for the amplification of the transgene (expected amplicon: 677 bp) and internal control region (expected amplicon: 324 bp) with GoTaq Green Master mix (Promega, Madison, WI, USA): oIMR8738 (L1‐Tg), 5′‐ATCACTGGAAGCGAGTCTGTCG‐3′; oIMR8739 (L1‐Tg), 5′‐TGCCCTTCTTGGGGTCCACCA‐3′; oIMR7338 (WT), 5′‐CTAGGCCACAGAATTGAAAGATCT‐3′; oIMR7339 (WT), 5′‐GTAGGTGGAAATTCTAGCATCATCC‐3′. The amplicons were electrophoretically separated on 2% agarose gel and analyzed with UV light.

The mice used in this study were males at 6‐12 weeks of age and were maintained on a standard diet and water ad libitum under a 12 h/12 h light/dark cycle (starting at 7:00), as described previously.^16^ Male mice from each litter were weaned and genotyped at 4 weeks of age and then fed a control‐fat diet (CFD) [CLEA Rodent Diet CE‐2, a standard diet for mice: CLEA Japan, Inc, Tokyo, Japan] for up to 6 weeks of age when the dietary administration was started in each randomly assigned group of mice. As a high‐fat diet (HFD) containing cholesterol, we used CE‐2 with 1% cholesterol, 0.5% cholic acid, and 10% palm oil (D15002: CLEA Japan, Inc). Diets containing ezetimibe (Sequoia Research Products Ltd., Pangbourne, UK) or IAXO101 (Innaxon, Tewkesbury, UK) at indicated concentrations were made by mixing powdered HFD with each substance before use. When we addressed mature adult mice, male mice at 17 weeks of age were subjected to a dietary study for 2 weeks. At the indicated time points, blood and/or bile specimens were taken immediately under no fasting conditions, and serum specimens were prepared as described previously .^17^ At necropsy, livers were excised and weighed, then rapidly frozen and stored in liquid nitrogen until further processing. Other specimens were stored at −80°C until use.

### 2.3 Sample size

Each experiment was designed to use the minimum number of mice or samples required to obtain informative results and sufficient material for subsequent studies. Although no statistical methods were used to pre‐determine sample size in vitro and in vivo analyses, based on preliminary results or an empirical approach we determined sufficient sample size. Samples that had undergone technical failure during processing were excluded from analyses. The numbers of biological replicates (*n*) are described in the figure legends.

### 2.4 Liver histology

Cryostat sections (5 μm) of the murine livers were cut from snap‐frozen tissues in hexanes with dry ice embedded in Tissue‐Tek OCT Compound (Sakura Finetek Japan Co., Ltd., Tokyo, Japan) and then air‐dried onto glass slides at room temperature. Cryosections of livers were stained by Oil Red O and counterstained with hematoxylin to visualize the lipid droplets. H&E and Oil Red O staining were performed according to the standard protocols.

### 2.5 Lipids extraction and biochemical measurements

Lipid extraction from liver samples was performed according to the well‐known Bligh and Dyer method.^18^ In brief, 800 μL of homogenized liver solution (50 mg of snap‐frozen liver/mL of distilled water) was mixed with 1 mL of chloroform and 2 mL of methanol, then well vortexed. After 10 minutes, 1 mL of chloroform was added to the mixture, and after blending, 1 mL of distilled water was added. After blending, the homogenate mixture was centrifuged at 1800× *g* for 10 minutes. After complete removal of the alcoholic (top) layer, 1 mL of the resulting chloroform (bottom) layer was transferred to a new glass tube, then evaporated to dryness under a stream of nitrogen. The resulting lipid extract was evaporated to dryness under a stream of nitrogen in a fresh glass tube, dissolved in isopropanol containing 10% (w/w) Triton X‐100, and subjected to measurements of hepatic cholesterol and TG. For quantitative calibration curves, standard samples containing known concentrations of cholesterol and TG were prepared in a similar manner. The concentrations of cholesterol and TG in each sample were measured using commercially available kits (summarized in Table 1) according to manufacturer's instructions.

### 2.6 Generation of and infection with adenoviruses

Using a ViraPower^TM^ Adenovial Gateway^TM^ Expression Kit (Invitrogen Carlsbad, CA), a recombinant adenovirus for expressing NPC1L1 (NCBI accession: NM_001101648), tagged with EGFP, was constructed and purified as described previously.^14^ The purified adenovirus was stored at −80°C until use. The resulting adenovirus titer was determined using an Adeno‐X^TM^ Rapid Titer Kit (Clontech Laboratories, Inc, Palo Alto, CA). To obtain transgenic mice transiently expressing NPC1L1, we intravenously administered the NPC1L1‐EGFP‐expressing adenoviruses (1 × 10^10^ ifu/20 g of body weight [BW]) into WT mice as described previously.^19^ As a control, mice were administered a recombinant adenovirus expressing EGFP.

### 2.7 Preparation of protein lysates

To make protein extracts, frozen livers were weighed and defrosted on ice, then homogenized (g of tissue/20 mL) using an ice‐cold Physcotron homogenizer (Microtec Co., Ltd., Chiba, Japan) in ice‐cold RIPA lysis buffer: 50 mmol/L Tris‐HCl, pH 7.4, 150 mmol/L NaCl, 0.1% sodium dodecyl sulfate (SDS), 0.5% sodium deoxycholate, 1% NP‐40, 1 mmol/L phenylmethylsulfonyl fluoride, and a Protease Inhibitor Cocktail for General Use (Nacalai Tesque, Kyoto, Japan). All protease inhibitors were added immediately prior to use. Crude lysates were incubated at 4ºC for 30 minutes with gentle rotation, before clarification by centrifugation at 20 000× *g* at 4°C for 30 minutes. The resulting supernatant was carefully collected in a new tube, and the protein concentration was determined by using the BCA Protein Assay Kit (Pierce, Rockford, IL) with BSA as a standard according to the manufacturer's protocol. The liver lysate samples were subjected to immunoblot analyses.

### 2.8 Immunoblotting

Immunoblot analyses were performed as described in our previous report^7^ with minor modifications. Briefly, liver lysate samples were separated by SDS‐PAGE and transferred to an Immobilon‐P PVDF membrane (Millipore Corp., Bedford, MA) by electroblotting at 15 V for 51 minutes. For blocking, the membrane was incubated in Tris‐buffered saline containing 0.05% Tween 20 and 3% BSA (TBST‐3%BSA). Blots were probed with appropriate antibodies (Table 1), and then the signals were visualized by a chemiluminescent method. All antibodies were used at 1:1000 (primary antibody) or 1:2000 (secondary antibody) dilution in TBST‐0.1%BSA for 1 hour at room temperature. After washing in TBST for 1 hour at room temperature, HRP‐dependent luminescence was developed with ECL^TM^ Prime Western Blotting Detection Reagent (GE Healthcare UK Ltd., Buckinghamshire, UK) and detected using a luminescent image analyzer (Bio‐Rad Laboratories, Tokyo, Japan).

### 2.9 Treatment with clodronate liposomes

For the long‐term depletion of macrophages in vivo, clodronate liposomes (Clophosome® (neutral): F70101C‐N, FormuMax Scientific, Palo Alto, CA, USA) or control liposomes (F70101‐N, FormuMax Scientific) were intravenously administrated (100 μL of liposome solution containing 10% sucrose and 20 mmol/L NaPO_4_ [pH 7.4]/20 g BW) in 6‐week‐old L1‐Tg mice at days 0, 3, 7, and 11. The clodronate liposome‐treated mice were fed a HFD for 2 weeks. Two treatment groups were studied: (a) control liposomes‐treated HFD‐fed L1‐Tg mice and (b) clodronate liposomes‐treated HFD‐fed L1‐Tg mice.

### 2.10 RNA extraction and qPCR

Total RNA was extracted from mouse livers using the RNA isoPlus^®^ Reagent (Takara Bio, Inc, Shiga, Japan), according to the manufacturer's protocol. Reverse transcriptional reaction and subsequent quantitative PCR (qPCR) using SYBR^®^ GreenER™ qPCR SuperMix Universal (Life Technologies, Tokyo, Japan) were performed as described previously.^14^ The sequences of the primers used are shown in Table 2.

**Table 2:** Primer sequences for qPCR analysis for each gene in *Mus musculus*

Symbol	Gene name	Sequence 5′ to 3′	
β‐actin	Actin, beta	F	AGATCAAGATCATTGCTCCTCCTG
		R	AACGCAGCTCAGTAACAGTCC
Ccl2	Chemokine (C‐C motif) ligand 2	F	GTGTTGGCTCAGCCAGATGC
		R	GACACCTGCTGCTGGTGATCC
F4/80	Adhesion G protein‐coupled receptor E1	F	CCCCAGTGTCCTTACAGAGTG
		R	GTGCCCAGAGTGGATGTCTC
Ho1	Heme oxygenase 1	F	ACATCGACAGCCCCACCAAGTTCAA
		R	CTGACGAAGTGACGCCATCTGTGAG
Tnf	Tumor necrosis factor	F	ATGAGAAGTTCCCAAATGGC
		R	CTCCACTTGGTGGTTTGCTA
Xbp1s	X‐box binding protein 1, spliced form	F	GCTGAGTCCGCAGCAGGTG
		R	GTGTCAGAGTCCATGGGAAGA
Xbp1u	X‐box binding protein 1, unspliced form	F	GAGTCCGCAGCACTCAGACT
		R	GTGTCAGAGTCCATGGGAAGA

F, forward; R, reverse. Primer sets for the detection of Xbp1 were derived from a previous report.^20^ The expression levels of each gene were normalized to those of β‐actin.

### 2.11 RNA‐expression profiling by microarray analysis

For microarray analysis, total RNA was purified using the RNeasy Micro Kit (Qiagen, Hilden, Germany) according to manufacturer's instructions. RNA integrity was evaluated using a Bioanalyzer 2100 (Agilent Technologies Inc, Santa Clara, CA). Total RNAs extracted from two or three mice were pooled, reverse‐transcribed to double‐stranded complementary DNA, and then in vitro transcribed to yield complementary RNA (cRNA) labeled with the fluorescent dye Cy3, according to the manufacturer's protocols. Cy3‐labeled cRNA was hybridized to the SurePrint G3 Mouse Gene Expression 8 × 60 K Microarray (Agilent Technologies) consisting of 62,976 spots. Three independent analyses were performed per group. The microarray was then washed using the Gene Expression Wash Pack (Agilent Technologies) and scanned with a DNA Microarray Scanner (G2565CA; Agilent Technologies). Scanner output images were normalized and digitalized using Feature Extraction software (Agilent Technologies) according to the Minimum Information About a Microarray Experiment (MIAME) guidelines.^21^ Independent three analyses were performed per each group. The difference index was calculated for each gene as (M1−M2)/(SD1+SD2), where M1 and M2 are the means normalized intensities for L1‐Tg and WT mice, respectively, and SD1 and SD2 are the standard deviations of the normalized intensities for each group. The threshold of expression change was set to a modulus of the difference index >0.7.

### 2.12 Functional analysis of microarray data

To better understand the biological meaning of the microarray results, functional analysis was performed by gene‐ontology (GO) analysis, as previously described.^22^ Briefly, genes were annotated with GO using an annotation file (gene2go.gz, updated on January 28, 2013) provided by the NCBI. Enrichment factors of differentially expressed genes were calculated for each GO. Statistical analysis was performed with Fisher's exact test based on a hypergeometric distribution, and GO terms with an enrichment factor ≥2, *nf* ≥4 and *P* < 0.01 were extracted. Microarray data obtained in this study were deposited in Gene Expression Omnibus (GEO) under accession code GSE110285.

### 2.13 Statistics

Unless otherwise noted, figures are presented as mean ± SEM. All statistical analyses were performed by using Excel 2013 (Microsoft Corp., Redmond, WA, USA) with Statcel3 add‐in software (OMS publishing Inc, Saitama, Japan). Different statistical tests were used for different experiments as described in the figure legends which include the numbers of biological replicates (*n*). When analyzing multiple groups, the similarity of variance between groups was compared using Bartlett's test. When passing the test for homogeneity of variance, a parametric Tukey–Kramer multiple‐comparison test was used; otherwise a non‐parametric Steel‐Dwass test was used. In the case of a single pair of quantitative data, after comparing the variances of a set of data by *F*‐test, unpaired Student's or Welch's *t *test was performed. Statistical significance was defined in terms of *P* values less than 0.05 or 0.01.

## 3 RESULTS

### 3.1 Hepatic NPC1L1 accelerated hepatic lipid accumulation resulting in steatosis

**Figure 1:**
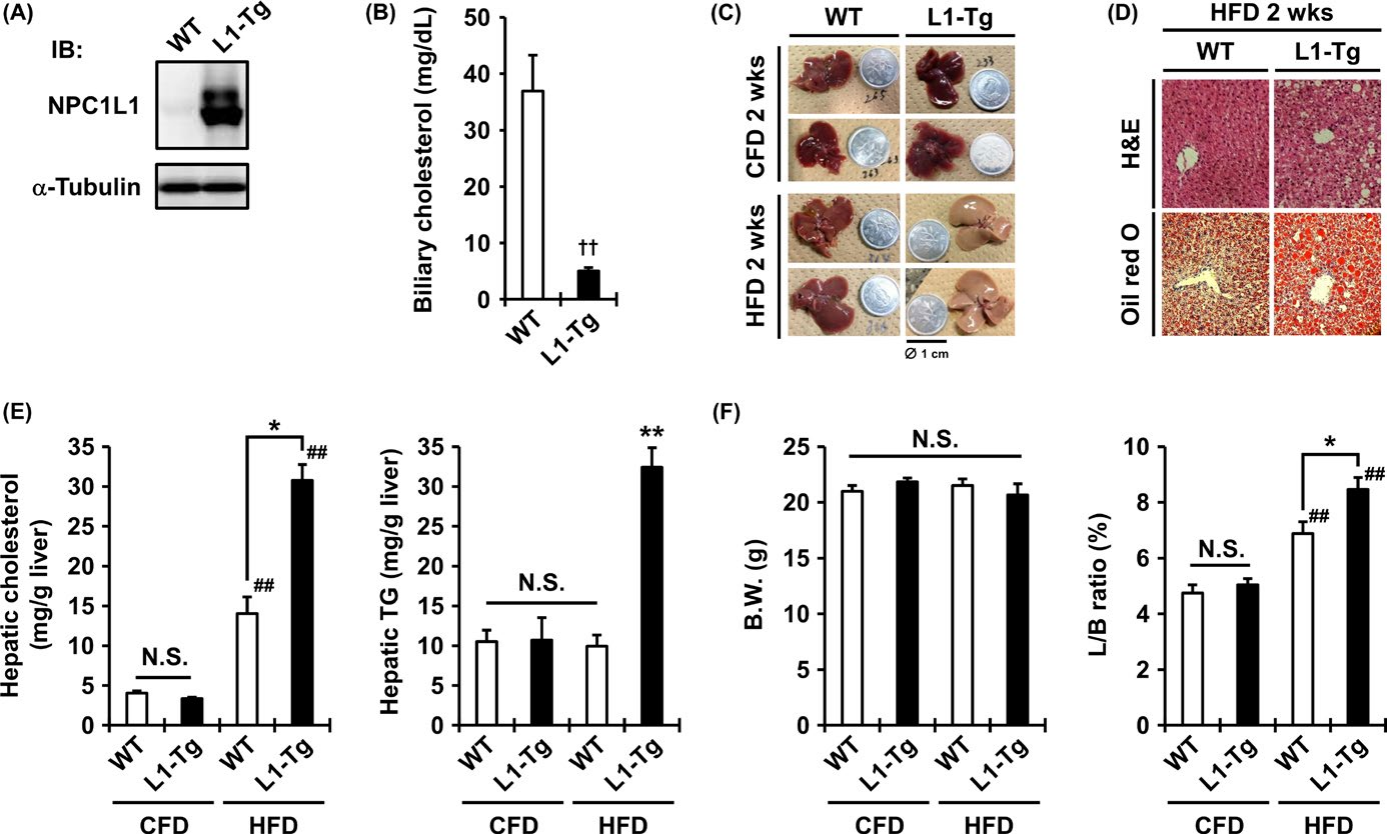
Hepatic NPC1L1‐mediated steatosis in the liver of L1‐Tg mice. A, Hepatic expression of the human NPC1L1 protein in L1‐Tg mice demonstrated by immunoblotting using an anti‐NPC1L1 antibody. α‐Tubulin, a loading control. B, Decrease of biliary cholesterol levels in L1‐Tg mice. Data are expressed as the mean ± SEM *n* = 6 (WT) and 7 (L1‐Tg). C, Photographic images of the livers of WT and L1‐Tg mice fed a control fat diet (CFD) or a high‐fat diet (HFD) for 2 weeks. The coin diameter was 1 cm. D, Hematoxylin and eosin (H&E) and Oil Red O staining of the livers of WT and L1‐Tg mice fed a HFD for 2 weeks. E and F, Hepatic cholesterol levels (*E*, *left*), hepatic triglyceride (TG) levels (*E*, *right*), body weight (BW) (*F*, *left*), and the ratios of liver weight to BW (L/B ratio) (*F*, *right*) in each group of mice fed a CFD or HFD for 2 weeks. Data are expressed as the mean ± SEM *n* = 11 (WT‐CFD) and 7 (the other groups). Statistical analyses for significant differences were performed using Bartlett's test, followed by a parametric Tukey–Kramer multiple‐comparison test (*E, right; F*) or a non‐parametric Steel–Dwass test (*E*, *left*) (##*P* < 0.01 vs CFD controls; **P < 0.01 *vs.* the other groups; *P < 0.05 among two groups; NS, not significantly different among groups) as well as a two‐sided *t *test (††*P* < 0.01).

To investigate the association of hepatic NPC1L1 with NAFLD risk, we first fed L1‐Tg mice with western feed characterized by high fat, including cholesterol (high‐fat diet: HFD). These L1‐Tg mice showed hepatic expression of human NPC1L1 driven by the liver‐specific ApoE promoter (Figure 1A) and exhibited lower biliary secretion of cholesterol than WT mice (Figure 1B). Additionally, the hepatic NPC1L1 level in L1‐Tg mice was approximately 86% of that in the human liver when these levels were normalized to the expression of RAP (hepatic chaperone protein) in each species.^6^ As mice have little expression of hepatic Npc1l1, L1‐Tg mice must be an appropriate in vivo model for the evaluation of the physiological impact of hepatic NPC1L1.

After 2 weeks of feeding with HFD, only the liver of L1‐Tg mice displayed remarkable changes suggestive of steatosis formation (Figure 1C). Hepatic lipid accumulation in L1‐Tg mice was confirmed by histological observations of liver sections (Figure 1D). Biochemical analyses revealed that the hepatic levels of cholesterol and TG in L1‐Tg mice on a HFD were significantly higher than those on a CFD (Figure 1E). In WT mice, HFD feeding moderately increased hepatic cholesterol levels, but did not affect hepatic TG levels. At necropsy, no significant differences in body weight were observed among all groups (Figure 1F), whereas the ratios of liver weight to body weight (L/B ratios) in L1‐Tg mice fed a HFD were significantly higher than those in other groups (Figure 1F), suggesting that liver enlargement was associated with fat accumulation. Considering the physiological role of hepatic NPC1L1 as a cholesterol re‐absorber from bile, the NPC1L1‐mediated increase of hepatic cholesterol seems to be followed by TG accumulation in the liver, resulting in the steatosis formation, which was only observed in L1‐Tg mice. Additionally, similar results were obtained in mature adult mice (Supplemental Figure S1).

### 3.2 Ezetimibe administration prevented the lipid accumulation in L1‐Tg mice with HFD

**Figure 2:**
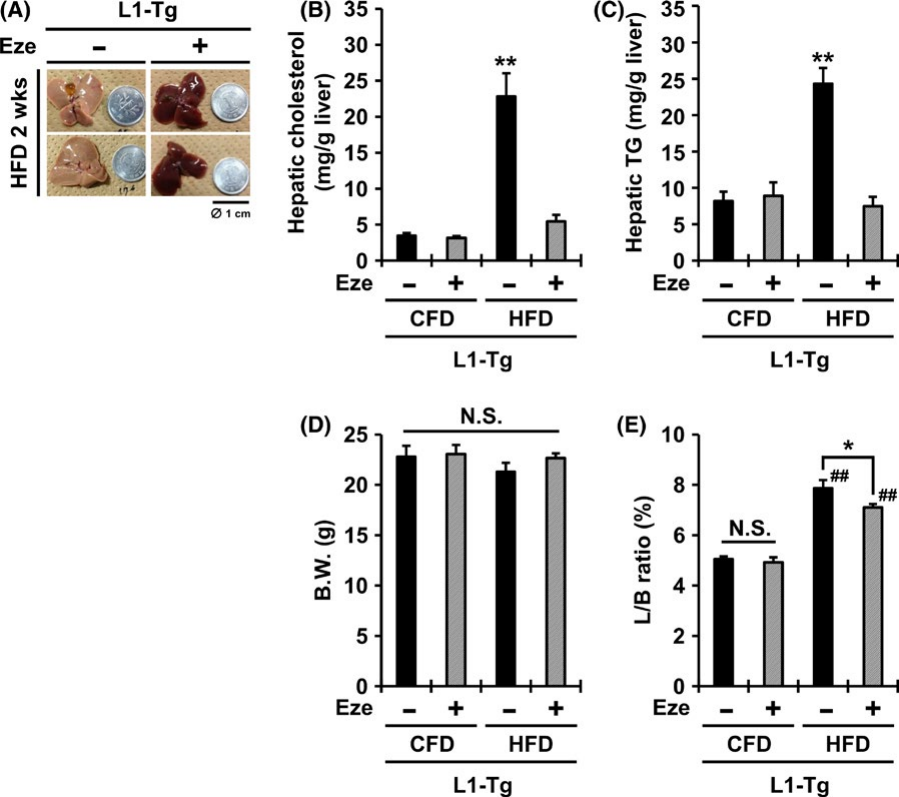
Complete prevention of steatosis in the livers of L1‐Tg mice fed a HFD by ezetimibe administration. A, Photographic images of the livers of L1‐Tg mice fed a high‐fat diet (HFD) containing ezetimibe (Eze) for 2 weeks. The coin diameter was 1 cm. B‐E, Hepatic cholesterol levels (*B*), hepatic triglyceride (TG) levels (*C*), body weight (BW) (*D*), and the ratios of liver weight to BW (L/B ratio) (*E*) in each group of mice. Data are expressed as the mean ± SEM *n* = 4 (non‐administered groups) and 7 (Eze‐administered groups). Statistical analyses for significant differences were performed using Bartlett's test, followed by a parametric Tukey–Kramer multiple‐comparison test (##*P* < 0.01 vs control fat diet (CFD) controls; **P < 0.01 *vs.* the other groups; *P < 0.05 among two groups; NS, not significantly different among groups).

To further examine the involvement of hepatic NPC1L1 in steatosis formation (Figure 1), we next administered the NPC1L1‐selective inhibitor ezetimibe to L1‐Tg mice for 2 weeks by mixing it in the mouse feed. Prior to the experiments, we confirmed that the presence of ezetimibe has little effect on the consumption of HFD (Supplemental Figure S2). The livers of L1‐Tg mice fed a HFD containing ezetimibe (16 μg/g diet) did not become steatotic (Figure 2A). Indeed, ezetimibe co‐administration suppressed the significant increases in the hepatic levels of cholesterol and TG in L1‐Tg mice fed a HFD (Figure 2B, C). At necropsy, no differences were found in the body weight of L1‐Tg mice among all groups (Figure 2D). However, in L1‐Tg mice fed a HFD, the L/B ratios in the ezetimibe‐administered group (7.1 ± 0.1%) were significantly lower than those in the non‐administered group (7.9 ± 0.3%) (Figure 2E), and comparable to those in WT mice fed a HFD (6.9 ± 0.4%) (Figure 1F). Ingestion of ezetimibe was supported by its pharmacological effect (Supplemental Figure S3). Integrated data of each hepatic parameter shown in Figures 1 and 2 are summarized in Supplemental Figure S4.

Moreover, to exclude the possibility that unexpected genetic alterations in L1‐Tg mice may have affect steatosis formation, we transiently expressed NPC1L1 in the liver of WT mice using an NPC1L1‐expressing adenovirus (L1‐Av) (Supplemental Figure S5). The levels of hepatic cholesterol and TG in L1‐Av mice fed a HFD were higher than those of control‐Av mice and these lipid accumulating phenotypes were attenuated by ezetimibe administration, which is consistent with our findings using L1‐Tg mice.

These results indicate that ezetimibe prevented the lipid accumulation in the livers of L1‐Tg mice fed a HFD, supporting the possibility that hepatic NPC1L1 exacerbates steatosis, and, thus, that ezetimibe could be useful in preventing steatosis.

### 3.3 Transcriptomics revealed hepatic elevation of immune‐response genes in L1‐Tg mice

Next, we performed a microarray analysis using WT and L1‐Tg mice fed a CFD to characterize basal differences in hepatic gene‐expression patterns, because we hypothesized that differing characteristics in the livers of WT and L1‐Tg mice could affect their potential responses to a HFD. The large amount of data generated was interpreted by GO analysis, based on functional categorization with GO terms. No significant differences were found in categories related to lipid homeostasis, such as GO:0006641 (TG metabolic process) and GO:0055088 (lipid homeostasis). However, differentially expressed genes between WT and L1‐Tg mice were enriched in 11 GO categories (Table 3). The most significant categories were “defense response to virus,” “response to virus,” and “innate immune response.” Immune response‐related genes were up‐regulated in the livers of L1‐Tg mice versus WT mice, even though the mice were not infected during the study. Based on these findings, we hypothesized that the apparent potent activation of the innate immune system could be involved as the initial trigger of the steatosis formation in L1‐Tg mice. In general, innate immune responses are transduced by some Toll‐like receptors (TLRs). Considering that recent findings have suggested the involvement of TLR4‐signaling in hepatic lipid accumulations,^23^, ^24^ we focused on TLR4 in subsequent experiments.

**Table 3:** Significantly enriched gene ontology categories in the livers of L1‐Tg mice as compared with WT mice

Categories	Number of genes		Enrichment factor	*P*‐value
	Up‐regulated	Down‐regulated		
Defense response to virus	18	1	7.03	<0.001
Response to virus	16	1	12.8	<0.001
Innate immune response	17	0	4.17	<0.001
Double‐stranded RNA binding	9	0	7.66	<0.001
Anchored to membrane	9	0	3.79	0.001
Immune response	9	0	2.75	0.006
Cellular response to interferon‐beta	7	0	13.3	<0.001
Negative regulation of viral genome replication	6	0	11.4	<0.001
2′‐5′oligoadenylate synthetase activity	5	0	40.0	<0.001
Cellular response to lipopolysaccharide	5	0	4.25	0.006
Glycoprotein binding	2	3	4.28	0.007

Enrichment factors for each GO term were defined as (nf/n)/(Nf/N), where nf is the number of flagged (differentially expressed) genes within a category, Nf is the total number of genes within the same category, n is the number of flagged genes in the entire microarray, and N is the total number of genes on the microarray.

### 3.4 A TLR4 antagonist blocked the exacerbation of hepatic NPC1L1‐dependent steatosis

**Figure 3:**
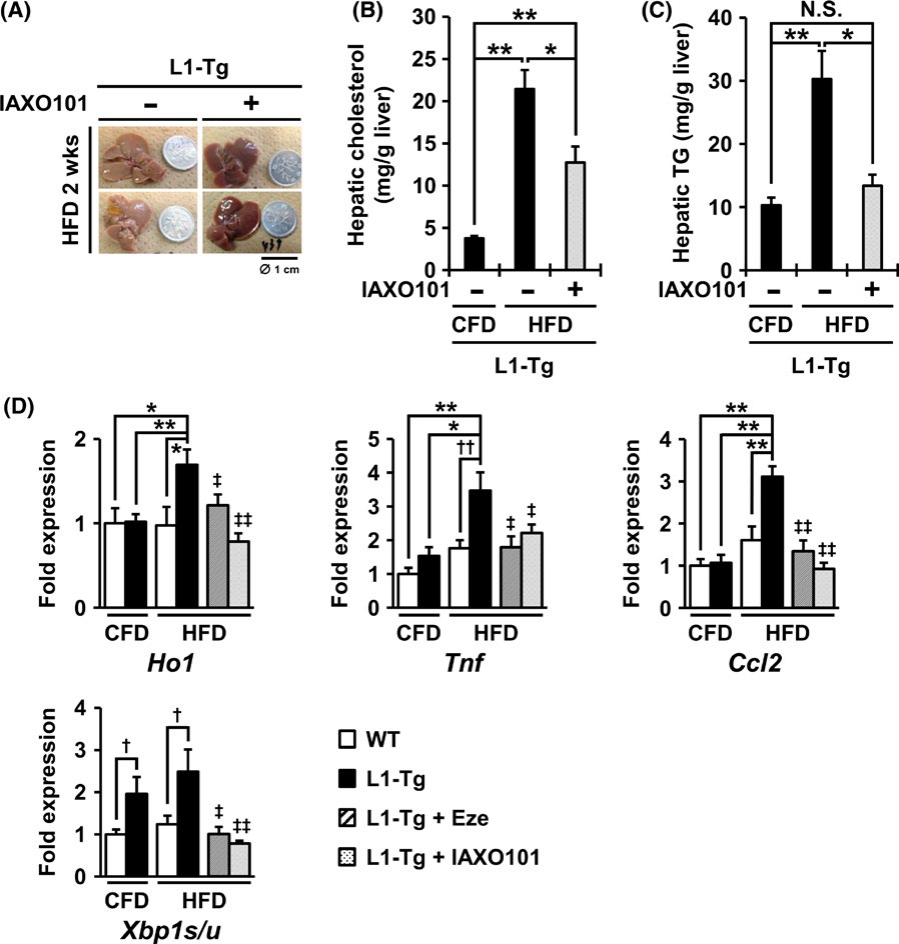
Prevention of hepatic NPC1L1‐mediated steatosis in L1‐Tg mice by administration of a TLR4 antagonist. A, Photographic images of the liver of L1‐Tg mice fed a high‐fat diet (HFD) containing IAXO101 (a Tlr4 antagonist) for 2 weeks. The coin diameter was 1 cm. B and C, Hepatic cholesterol levels (*B*) and hepatic triglyceride (TG) levels (*C*) in each group of L1‐Tg mice. Data are expressed as the mean ± SEM *n* = 5 (control fat diet (CFD) without IAXO101), 8 (HFD without IAXO101) and 9 (HFD with IAXO101). Statistical analyses for significant differences were performed according to Bartlett's test, followed by a non‐parametric Steel‐Dwass test (*P < 0.05; **P < 0.01; NS, not significantly different among groups). D, Expression changes of each hepatic gene in the livers of WT and L1‐Tg mice fed a CFD or HFD for 2 weeks. In the qRT‐PCR analyses, β‐actin mRNA was used as an internal control, and fold‐changes in the expression of each hepatic gene were normalized to the control (WT‐CFD) level. Data are expressed as the mean ± SEM *n* = 9 (CFD) and 6 (HFD) in WT mice; *n* = 16 (CFD), 15 (HFD), 8 (HFD with ezetimibe (Eze)), and 9 (HFD with IAXO101) in L1‐Tg mice. In the four non‐administered groups, statistical analyses for significant differences were performed using Bartlett's test, followed by a parametric Tukey–Kramer multiple‐comparison test (Ho1 and Ccl2) or a non‐parametric Steel‐Dwass test (Tnf and Xbp1s/u) (*P < 0.05; **P < 0.01), as well as a two‐sided *t*‐test (†*P* < 0.05; ††*P* < 0.01 among two groups). Regarding the Eze‐ or IAXO101‐administered groups, statistical analyses for significant differences were performed using a two‐sided *t* test (‡*P* < 0.05; ‡‡*P* < 0.01 *vs.* non‐administered L1‐Tg mice fed a HFD).

To examine the effect of Tlr4 inhibition on hepatic NPC1L1‐mediated steatosis, we fed L1‐Tg mice a HFD containing IAXO101 (12 μg/g of feed), a commercially available synthetic antagonist of TLR4 signaling, for 2 weeks. In the IAXO101‐administered group, steatosis was not observed (Figure 3A), and the hepatic levels of cholesterol and TG were lower than those in L1‐Tg mice fed a HFD (Figure 3B, C). In the livers of IAXO101‐administered L1‐Tg mice, despite the mild accumulation of cholesterol, little TG accumulated. This trend might imply that hepatic NPC1L1‐dependent re‐uptake of cholesterol followed by the activation of TLR4‐mediated cellular responses may be involved in steatosis formation.

We further explored the effect of IAXO101 administration on the mRNA levels of the following liver‐associated genes by qPCR. Hepatic mRNA expression of the oxidative stress marker (Ho1) and the typical cytokine/chemokine (Tnf and Ccl2) were higher than those of the other groups and were suppressed by both IAXO101 and ezetimibe administration (Figure 3D).

We obtained similar expression results for Xbp1s/u (a ratio of Xbp1 spliced‐form to its un‐spliced form) known as an endoplasmic reticulum (ER) stress marker (Figure 3D). Hitherto, an increase in the Xbp1s/u ratio was reportedly triggered by TLR4 stimulation in synergy with ER stress.^25^ Moreover, the Xbp1s/u ratios in the livers of L1‐Tg mice fed a CFD were higher than those of control mice fed a CFD (Figure 3D). These results may imply that hepatic NPC1L1 could stimulate Tlr4‐dependent cellular stress responses in the liver.

Additionally, depletion of hepatic macrophages by using clodronate liposomes relieved steatosis in L1‐Tg mice (Supplemental Figure S6). These results suggest the involvement of hepatic macrophages in steatosis formation in L1‐Tg mice, supporting a plausible link between Tlr4‐mediated reactions including macrophage activation and the exacerbation of steatosis in L1‐Tg mice.

### 3.5 Ezetimibe rescued steatosis in L1‐Tg mice continuously fed a HFD

**Figure 4:**
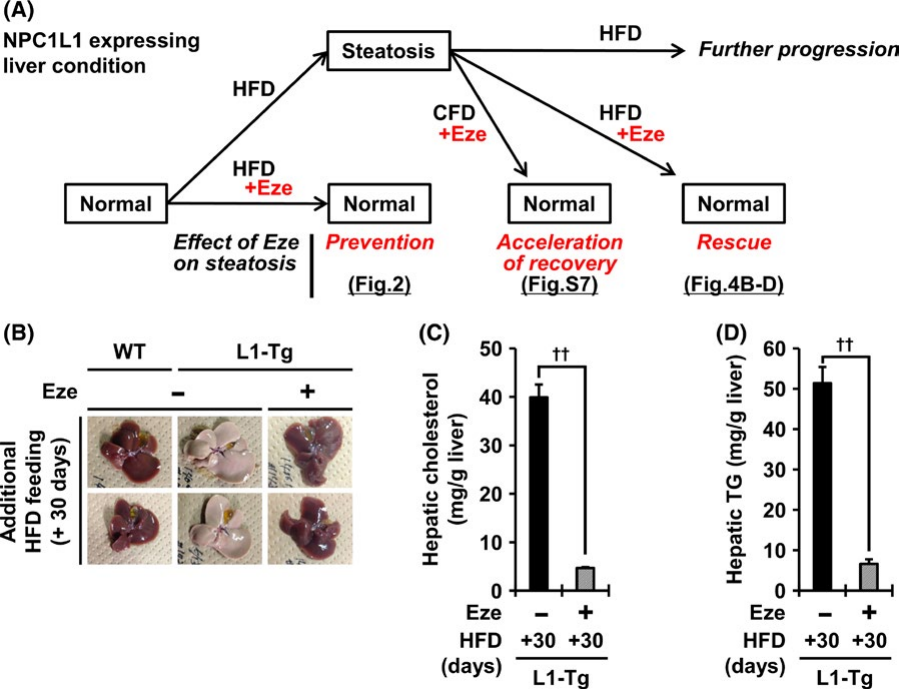
Complete rescue of hepatic NPC1L1‐mediated steatosis in L1‐Tg mice fed a HFD by post‐steatosis administration of ezetimibe with HFD. A, Schematic illustration of the beneficial effects of ezetimibe (Eze) in preventing and treating steatosis in L1‐Tg mice. B‐D, After high‐fat diet (HFD) feeding for 2 weeks, the mice were further fed a HFD containing Eze for 30 additional days. Photographic images of the livers of L1‐Tg mice fed maintained on a HFD containing Eze (*B*). While non‐Eze feeding aggregated steatosis in L1‐Tg mice, Eze rescued the livers of L1‐Tg mice from steatosis: that is the resulting lipid levels in the liver of L1‐Tg mice were almost comparable to those of WT mice whose livers never exhibited features of steatosis in this study (Supplemental Figure S4). Time‐dependent changes in the hepatic levels of cholesterol (*C*) and triglyceride (TG) (*D*) in each group of mice. Data are expressed as the mean ± SEM. Black bars, L1‐Tg mice without Eze, *n* = 7; shaded bars, L1‐Tg mice with Eze, n = 6. Statistical analyses for significant differences were performed using a *t* test (††*P* < 0.01, two‐sided).

Finally, to gain insight into the anti‐NAFLD efficacy of ezetimibe, we examined whether ezetimibe could rescue steatosis in L1‐Tg mice in two ways (Figure 4A).

First, L1‐Tg mice were fed a HFD for 2 weeks and then subjected to additional feeding with a CFD containing ezetimibe. Ten days after the extra‐feeding, L1‐Tg mice in the ezetimibe administrated‐group showed signs of recovery from steatosis (Supplemental Figure S7). Indeed, at both 10 and 30 days after initiating ezetimibe administration, the hepatic levels of cholesterol and TG in the ezetimibe‐administered group were lower than those in the non‐administered group. These results indicated that ezetimibe could inhibit the further progression of steatosis and accelerate the recovery from steatosis during CFD feeding.

Then, we examined whether ezetimibe could cure established steatosis under HFD feeding. L1‐Tg mice with steatosis were further fed a HFD containing ezetimibe for 30 days. Surprisingly, while lipid accumulation progressed in non‐administered group, the fatty livers of L1‐Tg mice in the ezetimibe‐administered group recovered to the control level (Figure 4B‐D, Supplemental Figure S4.). These results suggest that ezetimibe could not only prevent the progression of steatosis, but also reverse it without dietary restriction.

## 4 DISCUSSION

In this study, we identified hepatic NPC1L1 as a novel factor that exacerbates steatosis (Figures 1 and 2). We also demonstrated a new potency of ezetimibe, an NPC1L1‐selective inhibitor clinically used against dyslipidemia, as a preventive and curative drug for steatosis (Figures 2 and 4), which will be of importance in terms of drug repositioning. Thus, the pharmacological inhibition of NPC1L1 and its regulators are potential new targets of NAFLD therapy. Considering the physiological role of NPC1L1 in the liver, it seems that hepatic cholesterol incorporated from bile via this transporter could affect NAFLD progression. The detailed molecular basis underlying the early stage of hepatic NPC1L1‐mediated steatosis is an important research topic for future studies.

Our findings strongly suggest that hepatic NPC1L1 can exacerbate NAFLD, including steatosis in humans. Available information is limited, but a microarray transcriptomic data set from a previous study (GSE48452; human liver biopsy of different phases from control to NASH)^15^ may shed light on the latent link between NAFLD condition and hepatic NPC1L1‐expression levels. Re‐analysis of this data set showed that NPC1L1‐expression levels in the livers of patients with steatosis and NASH were higher than those in normal controls (Supplemental Figure S8), suggesting that subjects with higher levels of NPC1L1 may be more prone to NAFLD. However, it is also possible that NAFLD progression might have enhanced hepatic NPC1L1 expression. One plausible explanation for this interpretation could be positive regulation of NPC1L1 expression by HNF4α^26^ a transcriptional factor induced by hepatic injury. In either case, further epidemiological studies and mechanistic investigations of the association between NAFLD aggravation and the increased NPC1L1 hepatic levels are warranted.

The anti‐NAFLD effect of ezetimibe is the most important clinical implication of this study. Indeed, ezetimibe administration could not only prevent (Figure 2) but could also cure (Figure 4) hepatic NPC1L1‐mediated steatosis. Notably, despite starting after the steatosis formation, later administration of ezetimibe could rescue L1‐Tg mice with continuous HFD feeding (without dietary restriction) from steatosis, an initial stage of NAFLD progression (Figure 4B‐D). A series of our novel findings provides molecular evidence for the expected beneficial effects of ezetimibe on NAFLD/NASH treatment.^4^, ^12^ On the other hand, a clinical study referred to as the Mozart trial (24 weeks’ treatment with ezetimibe or placebo‐control) revealed that ezetimibe treatment in patients with NASH lowered liver fat by a small, but clinically unimportant, amount.^27^ Carefully considering this clinical information and our experimental findings, ezetimibe monotherapy might not improve the liver condition in NASH, but could be effective in treating and/or preventing early NAFLD conditions, at least steatosis. Although ezetimibe has been used in patients with NAFLD as a lipid‐lowering drug, its effect on the hepatic fatty condition in humans has not been fully evaluated. Additionally, previous reports pointed out that, despite several therapeutic trials of pharmacological agents, no highly effective treatment for NAFLD yet exists besides multifaceted lifestyle interventions such as diet, exercise, and behavioral counseling.^1^, ^28^ Therefore, further clinical studies are needed to examine whether ezetimibe alone or in combination with such interventions can exert therapeutic and/or protective effects on early steatosis in humans.

Regarding the potential efficacy of TLR4 inhibition on NAFLD, we showed that oral administration of a TLR4 antagonist prevented steatosis in L1‐Tg mice (Figure 3). Considering previous findings based on the studies using conventional or hepatic *Tlr4* KO mice that suggest that TLR4 in hepatocytes plays a pivotal role during the early progression of HFD‐induced NAFLD,^23^, ^24^ the presence of hepatic NPC1L1 might accelerate the Tlr4‐related exacerbation of steatosis. An important question for future studies is to determine whether TLR4 is a dependent or independent factor in the development of hepatic NPC1L1‐mediated steatosis. Given that intracellular accumulation of cholesterol could increase TLR4 proteins on the plasma membrane,^4^, ^29^ we can envision a possibility that hepatic NPC1L1‐mediated cholesterol re‐absorption might enhance a TLR4‐signaling in hepatocytes. Future study using further genetically engineered mice on the hepatic *Tlr4* KO background will help us in addressing these issues we noticed in this study via in vivo Tlr4 inhibition experiments.

Moreover, although the molecular basis remains to be elucidated, simultaneous inhibition of hepatic NPC1L1 and TLR4 might have greater therapeutic effect in treating steatosis versus monotherapy with either inhibitor. Because a link between TLR4 activation and NAFLD progression has begun to be understood, the use of TLR4 inhibitor as an anti‐NAFLD agent should attract interest. Considering the therapeutic importance of down‐modulation of TLR4 signaling in patients with such life‐threatening diseases as sepsis and inflammatory bowel disease, pharmaceutical companies and laboratories have developed numerous TLR4 antagonists; some of them are either in or have completed clinical trials.^30^ Although two well‐known TLR4 antagonists, TAK‐242 and Eritoran, could not reach the market due, not to safety considerations, but to an inadequate clinical outcome (about 28‐day all‐cause mortality) in a phase 3 study of patients with severe sepsis,^31^, ^32^ other candidates with increased potency have entered clinical trials. To the best of our knowledge, it remains to be elucidated whether such TLR4 antagonists, including those that were dropped, could have therapeutic efficacy in treating human NAFLD. Future work should therefore be conducted to evaluate this clinically important issue with consideration of the possibility that the combined use of ezetimibe could boost the expected outcome of TLR4 antagonists.

In conclusion, we demonstrated that hepatic NPC1L1 can exacerbate steatosis with possibility that biliary‐derived cholesterol is involved in NAFLD progression. Our findings provides a deeper understanding of NAFLD mechanisms. Moreover, the anti‐NAFLD effect of ezetimibe, as evidenced by the complete prevention and rescue of steatosis, together with the potential medicinal property of the TLR4 antagonist, could contribute to the development of new therapeutic strategies for this global health problem.

## CONFLICT OF INTEREST

The authors declare that they have no conflict of interest.

## AUTHOR CONTRIBUTIONS

Y. Toyoda and T. Takada conceived and designed the study, interpreted the data, and wrote the manuscript; Y. Toyoda and F. Tumura performed the experimental work and analyzed the data; M. Umezawa and K. Takeda were responsible for the microarray GO analysis; Y. Yamanashi provided precious materials; H. Suzuki supervised the study and assisted in preparing the manuscript.

## References

[1] DeWeerdt S . Disease progression: divergent paths. Nature. 2017;551:S92‐S93. 10.1038/d41586-017-06925-2 29168825

[2] Wree A , Broderick L , Canbay A , Hoffman HM , Feldstein AE . From NAFLD to NASH to cirrhosis‐new insights into disease mechanisms. Nat Rev Gastroenterol. Hepatol. 2013;10:627‐636. 23958599 10.1038/nrgastro.2013.149

[3] Hebbard L , George J . Animal models of nonalcoholic fatty liver disease. Nat Rev Gastroenterol Hepatol. 2011;8:35‐44. 21119613 10.1038/nrgastro.2010.191

[4] Musso G , Gambino R , Cassader M . Cholesterol metabolism and the pathogenesis of non‐alcoholic steatohepatitis. Prog Lipid Res. 2013;52:175‐191. 23206728 10.1016/j.plipres.2012.11.002

[5] Mari M , Caballero F , Colell A , et al. Mitochondrial free cholesterol loading sensitizes to TNF‐ and Fas‐mediated steatohepatitis. Cell Metab. 2006;4:185‐198. 16950136 10.1016/j.cmet.2006.07.006

[6] Temel RE , Tang W , Ma Y , et al. Hepatic Niemann‐Pick C1‐like 1 regulates biliary cholesterol concentration and is a target of ezetimibe. J Clin Invest. 2007;117:1968‐1978. 17571164 10.1172/JCI30060 PMC1888567

[7] Yamanashi Y , Takada T , Shoda J , Suzuki H . Novel function of Niemann‐Pick C1‐like 1 as a negative regulator of Niemann‐Pick C2 protein. Hepatology. 2012;55:953‐964. 22095670 10.1002/hep.24772

[8] Altmann SW , Davis HR Jr , Zhu LJ , et al. Niemann‐Pick C1 Like 1 protein is critical for intestinal cholesterol absorption. Science. 2004;303:1201‐1204. 14976318 10.1126/science.1093131

[9] Garcia‐Calvo M , Lisnock J , Bull HG , et al. The target of ezetimibe is Niemann‐Pick C1‐Like 1 (NPC1L1). Proc Natl Acad Sci U S A. 2005;102:8132‐8137. 15928087 10.1073/pnas.0500269102 PMC1149415

[10] Baigent C , Landray MJ , Reith C , et al. The effects of lowering LDL cholesterol with simvastatin plus ezetimibe in patients with chronic kidney disease (Study of Heart and Renal Protection): a randomised placebo‐controlled trial. Lancet. 2011;377:2181‐2192. 21663949 10.1016/S0140-6736(11)60739-3 PMC3145073

[11] Cannon CP , Blazing MA , Giugliano RP , et al. Ezetimibe Added to Statin Therapy after Acute Coronary Syndromes. N Engl J Med. 2015;372:2387‐2397. 26039521 10.1056/NEJMoa1410489

[12] Ahmed MH , Byrne CD . Ezetimibe as a potential treatment for non‐alcoholic fatty liver disease: is the intestine a modulator of hepatic insulin sensitivity and hepatic fat accumulation? Drug Discov Today. 2010;15:590‐595. 20601094 10.1016/j.drudis.2010.06.007

[13] Musso G , Gambino R , Cassader M , Pagano G . A meta‐analysis of randomized trials for the treatment of nonalcoholic fatty liver disease. Hepatology. 2010;52:79‐104. 20578268 10.1002/hep.23623

[14] Toyoda Y , Takada T , Miyata H , Ishikawa T , Suzuki H . Regulation of the axillary osmidrosis‐associated ABCC11 protein stability by N‐linked glycosylation: effect of glucose condition. PLoS ONE. 2016;11:e0157172. 27281343 10.1371/journal.pone.0157172 PMC4900533

[15] Ahrens M , Ammerpohl O , von Schonfels W , et al. DNA methylation analysis in nonalcoholic fatty liver disease suggests distinct disease‐specific and remodeling signatures after bariatric surgery. Cell Metab. 2013;18:296‐302. 23931760 10.1016/j.cmet.2013.07.004

[16] Takada T , Yamanashi Y , Konishi K , et al. NPC1L1 is a key regulator of intestinal vitamin K absorption and a modulator of warfarin therapy. Sci Transl Med. 2015;7:275ra223. 10.1126/scitranslmed.3010329 25696002

[17] Toyoda Y , Takada T , Suzuki H . Halogenated hydrocarbon solvent‐related cholangiocarcinoma risk: biliary excretion of glutathione conjugates of 1,2‐dichloropropane evidenced by untargeted metabolomics analysis. Sci Rep. 2016;6:24586. 27087417 10.1038/srep24586 PMC5263858

[18] Bligh EG , Dyer WJ . A rapid method of total lipid extraction and purification. Can J Biochem Physiol. 1959;37:911‐917. 13671378 10.1139/o59-099

[19] Toyoda Y , Takada T , Gomi T , Nakagawa H , Ishikawa T , Suzuki H . Clinical and molecular evidence of ABCC11 Protein expression in axillary apocrine glands of patients with axillary osmidrosis. Int J Mol Sci. 2017;18:417. 10.3390/ijms18020417 PMC5343951 28212277

[20] Villeneuve J , Lepreux S , Mulot A , et al. A protective role for CD154 in hepatic steatosis in mice. Hepatology. 2010;52:1968‐1979. 21064031 10.1002/hep.23935

[21] Brazma A , Hingamp P , Quackenbush J , et al. Minimum information about a microarray experiment (MIAME)‐toward standards for microarray data. Nat Genet. 2001;29:365‐371. 11726920 10.1038/ng1201-365

[22] Hori H , Umezawa M , Uchiyama M , Niki R , Yanagita S , Takeda K . Effect of high‐fat diet prior to pregnancy on hepatic gene expression and histology in mouse offspring. J Perinat Med. 2014;42:83‐91. 23974043 10.1515/jpm-2013-0091

[23] Li L , Chen L , Hu L , et al. Nuclear factor high‐mobility group box1 mediating the activation of Toll‐like receptor 4 signaling in hepatocytes in the early stage of nonalcoholic fatty liver disease in mice. Hepatology. 2011;54:1620‐1630. 21809356 10.1002/hep.24552

[24] Jia L , Vianna CR , Fukuda M , et al. Hepatocyte Toll‐like receptor 4 regulates obesity‐induced inflammation and insulin resistance. Nat Commun. 2014;5:3878. 24815961 10.1038/ncomms4878 PMC4080408

[25] Martinon F , Chen X , Lee AH , Glimcher LH . TLR activation of the transcription factor XBP1 regulates innate immune responses in macrophages. Nat Immunol. 2010;11:411‐418. 20351694 10.1038/ni.1857 PMC3113706

[26] Iwayanagi Y , Takada T , Suzuki H . HNF4alpha is a crucial modulator of the cholesterol‐dependent regulation of NPC1L1. Pharm Res. 2008;25:1134‐1141. 18080173 10.1007/s11095-007-9496-9

[27] Loomba R , Sirlin CB , Ang B , et al. Ezetimibe for the treatment of nonalcoholic steatohepatitis: assessment by novel magnetic resonance imaging and magnetic resonance elastography in a randomized trial (MOZART trial). Hepatology. 2015;61:1239‐1250. 25482832 10.1002/hep.27647 PMC4407930

[28] Dowman JK , Armstrong MJ , Tomlinson JW , Newsome PN . Current therapeutic strategies in non‐alcoholic fatty liver disease. Diabetes Obes Metab. 2011;13:692‐702. 21449949 10.1111/j.1463-1326.2011.01403.x

[29] Musso G . Ezetimibe in the balance: can cholesterol‐lowering drugs alone be an effective therapy for NAFLD? Diabetologia. 2014;57:850‐855. 24554006 10.1007/s00125-014-3192-1

[30] Patra MC , Choi S . Recent progress in the development of Toll‐like receptor (TLR) antagonists. Expert Opin Ther Pat. 2016;26:719‐730. 27136061 10.1080/13543776.2016.1185415

[31] Opal SM , Laterre P‐F , Francois B , et al. Effect of eritoran, an antagonist of MD2‐TLR4, on mortality in patients with severe sepsis: the ACCESS randomized trial. JAMA. 2013;309:1154‐1162. 23512062 10.1001/jama.2013.2194

[32] Rice TW , Wheeler AP , Bernard GR , et al. A randomized, double‐blind, placebo‐controlled trial of TAK‐242 for the treatment of severe sepsis. Crit Care Med. 2010;38:1685‐1694. 20562702 10.1097/CCM.0b013e3181e7c5c9

