# Supplementary material for: Identification of hepatic NPC1L1 as an NAFLD risk factor evidenced by ezetimibe‐mediated steatosis prevention and recovery
Source: FASEB Bioadv. 2019 Feb 13;1(5):283–95. doi: 10.1096/fba.2018-00044 (PMC6996404; doi:10.1096/fba.2018-00044)
Supplement: Supplementary file 1 [file FBA2-1-283-s001.pdf]

Identification of hepatic NPC1L1 as an NAFLD-risk factor evidenced by ezetimibe-mediated steatosis prevention and recovery

Toyoda Y., Takada T. *et al.*

Supplemental Data

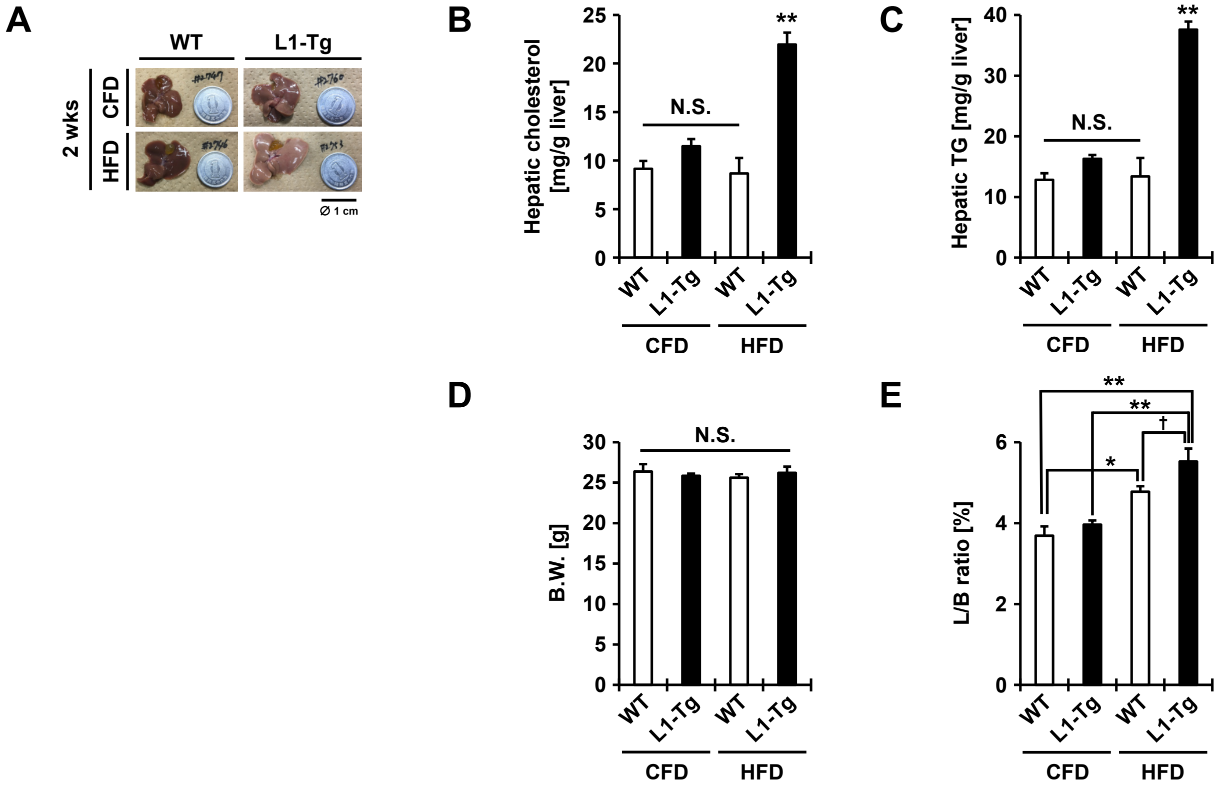

Fig. S1. Hepatic NPC1L1-dependent steatosis in mature adult L1-Tg mice fed a HFD.
Male mice at 17 weeks of age, which were maintained with a standard diet before the start of dietary administration, were studied. **(A)** Photographic images of the livers of WT and L1-Tg mice fed a control fat diet (CFD) or a high-fat diet (HFD) for two weeks. The coin diameter was one cm. **(B–E)** Hepatic cholesterol levels (B), hepatic triglyceride (TG) levels (C), body weight (B.W.) (D), and the ratios of liver weight to B.W. (L/B ratio) (E) in each group of mice. Data are expressed as the mean ± SEM. $n = 5$ (L1-Tg fed a HFD) and 4 (the other groups). Statistical analyses for significant differences were performed using Bartlett's test, followed by a parametric Tukey–Kramer multiple-comparison test (**, $P < 0.01$ *vs.* the other groups or indicated two groups; *, $P < 0.05$ among two groups; N.S., not significantly different among groups) or a one-sided *t*-test (†, $P < 0.05$).
