# Supplementary material for: Identification of hepatic NPC1L1 as an NAFLD risk factor evidenced by ezetimibe‐mediated steatosis prevention and recovery
Source: FASEB Bioadv. 2019 Feb 13;1(5):283–95. doi: 10.1096/fba.2018-00044 (PMC6996404; doi:10.1096/fba.2018-00044)
Supplement: Supplementary file 2 [file FBA2-1-283-s002.pdf]

Identification of hepatic NPC1L1 as an NAFLD-risk factor evidenced by ezetimibe-mediated steatosis prevention and recovery

Toyoda Y., Takada T. *et al.*

Supplemental Data

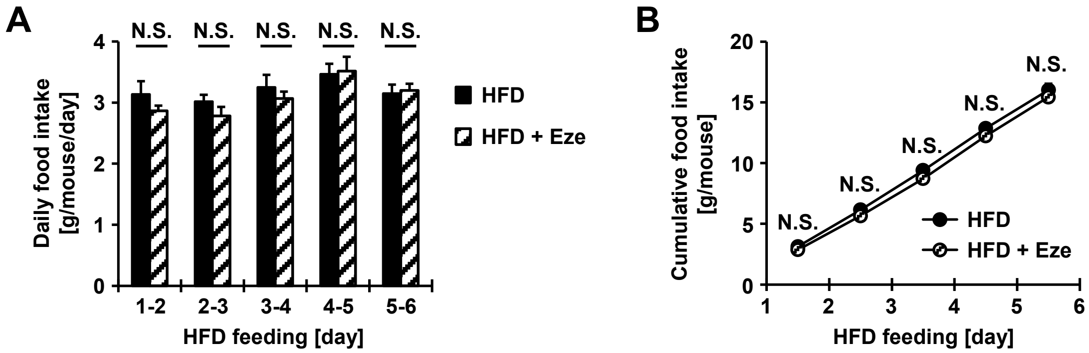

Fig. S2. Effect of ezetimibe on food intake of male mice fed a HFD.
Daily (A) and cumulative (B) intake of a high-fat diet (HFD) with or without ezetimibe (Eze) showed that the presence of ezetimibe did not affect the intake. In brief, male mice at 6 weeks of age were randomly assigned by body weight into two groups and caged individually at day 0. The control group received a HFD; another group was given a HFD containing ezetimibe. Each food provided was pre-weighed. From day 1, food intake was measured daily for 5 days. For this purpose, the amount of food remained, including any on the bottom of the cages as much as possible, was recorded per 24 hours. Intake amount was calculated by subtracting the weight of food remained from that provided. Data are expressed as the mean ± SEM, n = 6. Where vertical bars are not shown, the SEM is within the limits of the closed symbols. A two factor repeated measures ANOVA showed no significant group (HFD *vs.* HFD with Eze) × feeding time interaction and no effect of ezetimibe on the daily and cumulative amount of food intake, P = 0.396 (A) and 0.197 (B), respectively. Statistical analyses for significant differences were performed using a two-sided t-test (N.S., not significantly different among two groups).
