# Supplementary material for: Identification of hepatic NPC1L1 as an NAFLD risk factor evidenced by ezetimibe‐mediated steatosis prevention and recovery
Source: FASEB Bioadv. 2019 Feb 13;1(5):283–95. doi: 10.1096/fba.2018-00044 (PMC6996404; doi:10.1096/fba.2018-00044)
Supplement: Supplementary file 3 [file FBA2-1-283-s003.pdf]

Identification of hepatic NPC1L1 as an NAFLD-risk factor evidenced by ezetimibe-mediated steatosis prevention and recovery

Toyoda Y., Takada T. *et al.*

Supplemental Data

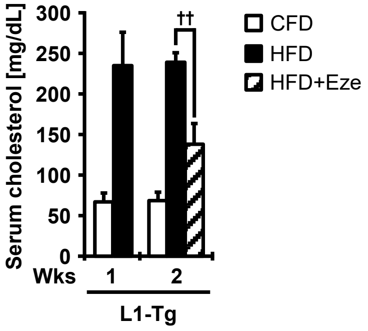

Fig. S3. Serum levels of cholesterol in L1-Tg mice fed a CFD or a HFD in the absence or presence of ezetimibe.

CFD, control fat diet; HFD, high-fat diet; Eze, ezetimibe. Data are expressed as the mean ± SEM. n = 7 (CFD) and 7 (HFD) at one week; 10 (CFD), 8 (HFD), and 4 (HFD with Eze) at two weeks. Statistical analyses for significant differences were performed using a two-sided t-test (††, $P < 0.01$).
