# Supplementary material for: Identification of hepatic NPC1L1 as an NAFLD risk factor evidenced by ezetimibe‐mediated steatosis prevention and recovery
Source: FASEB Bioadv. 2019 Feb 13;1(5):283–95. doi: 10.1096/fba.2018-00044 (PMC6996404; doi:10.1096/fba.2018-00044)
Supplement: Supplementary file 4 [file FBA2-1-283-s004.pdf]

Identification of hepatic NPC1L1 as an NAFLD-risk factor evidenced by ezetimibe-mediated steatosis prevention and recovery

Toyoda Y., Takada T. *et al.*

Supplemental Data

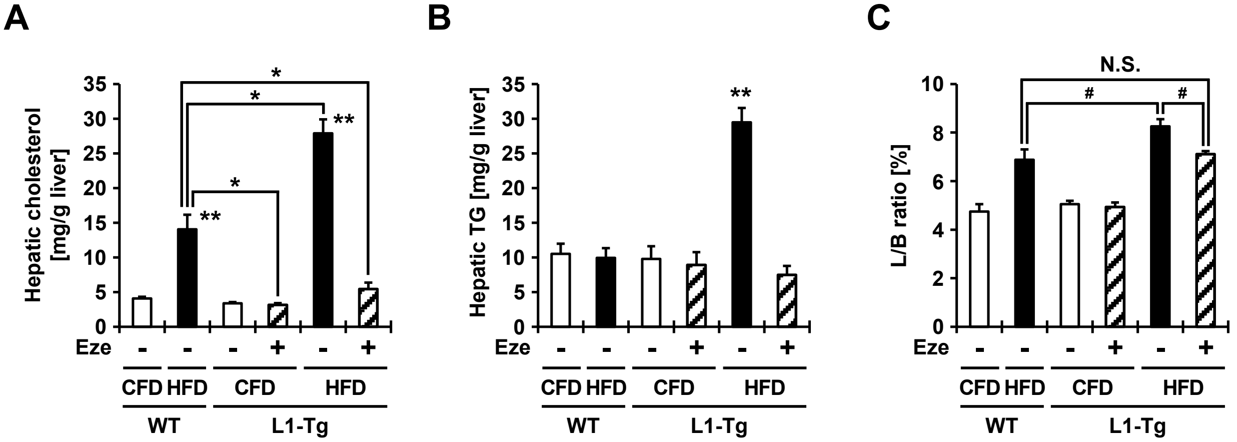

Fig. S4. Hepatic NPC1L1-mediated steatosis in the liver of L1-Tg mice and its complete prevention by ezetimibe administration.

Data from Figs. 1 and 2 are integrated and analyzed. **(A)** Hepatic cholesterol levels, **(B)** hepatic triglyceride (TG) levels, and **(C)** the ratios of liver weight to body weight (L/B ratio) in each group of mice fed a control fat diet (CFD) or high fat diet (HFD) with or without ezetimibe (Eze) for two weeks. Data are expressed as the mean ± SEM. $n = 11$ (WT-CFD, Tg-CFD or HFD without Eze) and 7 (the other three groups). Statistical analyses for significant differences in all groups were performed using Bartlett's test, followed by a parametric Tukey–Kramer multiple-comparison test (A and C) or a non-parametric Steel–Dwass test (B) (**, $P < 0.01$ vs. the other groups; *, $P < 0.05$ among two groups). In (C), each group fed a CFD was significantly different from each group fed a HFD (Steel–Dwass test for all the groups). #, $P < 0.05$ among two groups; N.S., not significantly different among groups (Steel–Dwass test for the three groups fed a HFD).
