# Supplementary material for: Identification of hepatic NPC1L1 as an NAFLD risk factor evidenced by ezetimibe‐mediated steatosis prevention and recovery
Source: FASEB Bioadv. 2019 Feb 13;1(5):283–95. doi: 10.1096/fba.2018-00044 (PMC6996404; doi:10.1096/fba.2018-00044)
Supplement: Supplementary file 5 [file FBA2-1-283-s005.pdf]

Identification of hepatic NPC1L1 as an NAFLD-risk factor evidenced by ezetimibe-mediated steatosis prevention and recovery

Toyoda Y., Takada T. *et al.*

Supplemental Data

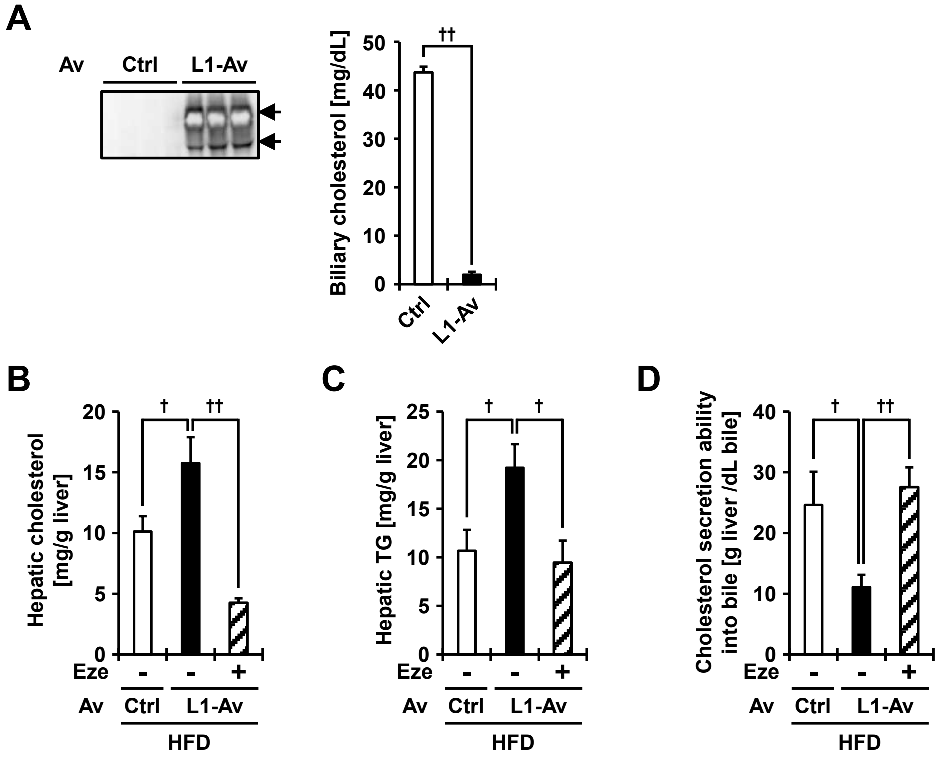

Fig. S5. Transiently expressed hepatic NPC1L1-mediated steatosis in adenovirus-infected mice fed a HFD and its prevention by ezetimibe administration.
(A) Hepatic expression of functional human NPC1L1 protein in EGFP-tagged NPC1L1 (L1-EGFP) expressing adenovirus infected mice (L1-Av mice) fed a control fat diet for five days. Immunoblotting was performed using an anti-EGFP antibody (*left panel*). EGFP-expressing adenovirus were used for control experiments (Ctrl). Arrows indicate NPC1L1-EGFP. The bar chart shows lower levels of biliary cholesterol in L1-Av mice ($n = 4$) than in Ctrl-Av mice ($n = 4$) (*right panel*). **(B–D)** Hepatic cholesterol levels (B), hepatic triglyceride (TG) levels (C), and hepatic ability in biliary cholesterol secretion (D) in each group of Av mice. After adenovirus infection, the Av mice were fed a high-fat diet (HFD) in the absence or presence of ezetimibe (Eze) for five days. The levels of hepatic cholesterol and TG in L1-Av mice were higher than those of Ctrl-Av mice; the lipid-accumulating phenotype in the livers of L1-Av mice was attenuated by ezetimibe administration (B and C). At necropsy, the hepatic ability of cholesterol secretion into bile (defined as the ratio of the cholesterol level in bile to that in the liver) in the L1-Av mice fed a HFD was lower than that in Ctrl-Av mice and was reversed to the control level by ezetimibe (D), suggesting that hepatic NPC1L1 was functionally expressed during the feeding. Data are expressed as the mean ± SEM. $n = 4$ (Ctrl-Av mice), 6 (L1-Av mice without Eze), and 4 (L1-Av mice with Eze). Statistical analyses for significant differences were performed using a one-sided *t*-test (†, $P < 0.05$; ††, $P < 0.01$).
