# Supplementary material for: Identification of hepatic NPC1L1 as an NAFLD risk factor evidenced by ezetimibe‐mediated steatosis prevention and recovery
Source: FASEB Bioadv. 2019 Feb 13;1(5):283–95. doi: 10.1096/fba.2018-00044 (PMC6996404; doi:10.1096/fba.2018-00044)
Supplement: Supplementary file 6 [file FBA2-1-283-s006.pdf]

Identification of hepatic NPC1L1 as an NAFLD-risk factor evidenced by ezetimibe-mediated steatosis prevention and recovery

Toyoda Y., Takada T. *et al.*

Supplemental Data

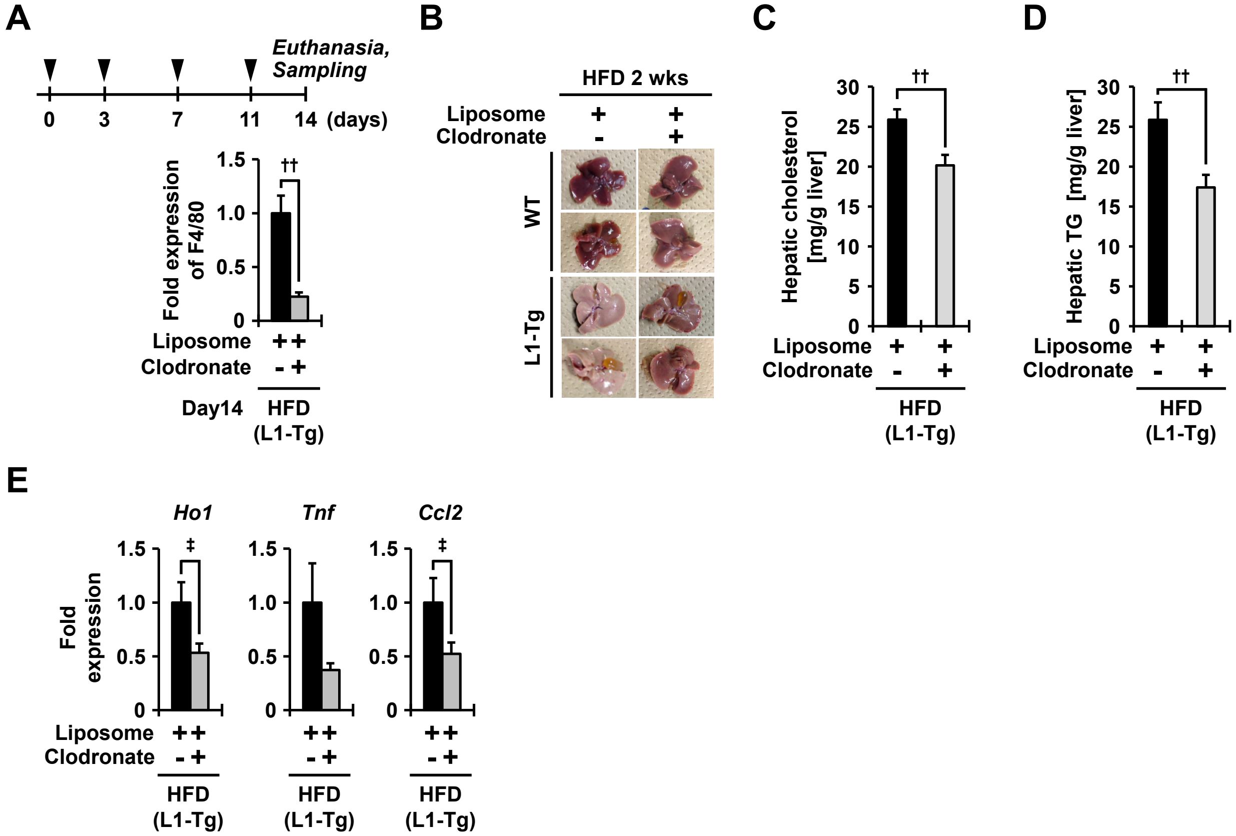

Fig. S6. Attenuation of hepatic NPC1L1-mediated steatosis in L1-Tg mice by macrophage depletion. **(A)** Successful depletion of hepatic macrophages by clodronate liposomes. *Upper panel*, time schedule of administration. Arrow heads indicates the intravenously administration of clodronate liposomes. *Lower panel*, hepatic mRNA levels of the macrophage marker gene, F4/80. **(B)** Photographic images of the livers of WT and L1-Tg mice fed a high-fat diet (HFD) under the macrophage-depleted condition for two weeks. **(C and D)** Lowering effect of macrophage depletion on the hepatic levels of cholesterol (C) and triglyceride (TG) (D) in L1-Tg mice fed a HFD for two weeks. **(E)** The effect of macrophage depletion on the mRNA levels of hepatic genes in L1-Tg mice fed a HFD for two weeks. Fold-changes in expression levels were normalized to the control level. Data are expressed as the mean ± SEM. n = 7 (day 14, two weeks). Statistical analyses for significant differences were performed using a t-test (††, $P < 0.01$, two-sided; ‡, $P < 0.05$, one-sided).
