# Supplementary material for: Identification of hepatic NPC1L1 as an NAFLD risk factor evidenced by ezetimibe‐mediated steatosis prevention and recovery
Source: FASEB Bioadv. 2019 Feb 13;1(5):283–95. doi: 10.1096/fba.2018-00044 (PMC6996404; doi:10.1096/fba.2018-00044)
Supplement: Supplementary file 7 [file FBA2-1-283-s007.pdf]

Identification of hepatic NPC1L1 as an NAFLD-risk factor evidenced by ezetimibe-mediated steatosis prevention and recovery

Toyoda Y., Takada T. *et al.*

Supplemental Data

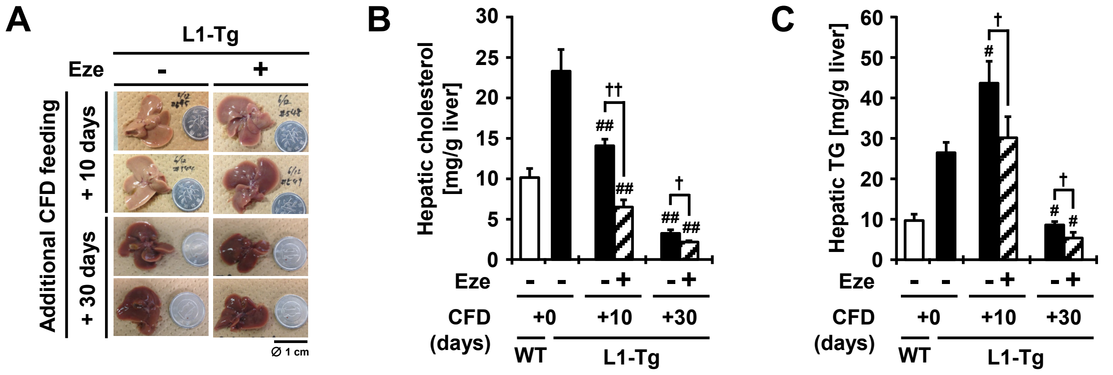

Fig. S7. Accelerated recovery from hepatic NPC1L1-mediated steatosis in L1-Tg mice by the post-steatosis administration of CFD containing ezetimibe.

Two weeks after high-fat diet (HFD) feeding to induce steatosis formation, L1-Tg mice were fed a control fat diet (CFD) containing ezetimibe (Eze) for an additional 10 or 30 days. **(A)** Photographic images of the livers of L1-Tg mice fed an additional CFD containing Eze. The coin diameter was one cm. **(B and C)** Time-dependent changes in the hepatic levels of cholesterol (B) and triglyceride (TG) (C) in each group of mice. Data are expressed as the mean ± SEM. White bars, WT, $n = 6$; black bars, L1-Tg mice without Eze, $n = 6$ (+0 day), 8 (+10 days), and 6 (+30 days); shaded bars, L1-Tg mice with Eze, $n = 9$ (+10 days) and 4 (+30 days). Statistical analyses for significant differences in L1-Tg mice groups were performed using Bartlett's test, followed by a Dunnett test (#, $P < 0.05$; ##, $P < 0.01$ *vs.* control, +0 day), as well as a one-sided *t*-test (†, $P < 0.05$; ††, $P < 0.01$ among two groups).
