# Supplementary material for: Identification of hepatic NPC1L1 as an NAFLD risk factor evidenced by ezetimibe‐mediated steatosis prevention and recovery
Source: FASEB Bioadv. 2019 Feb 13;1(5):283–95. doi: 10.1096/fba.2018-00044 (PMC6996404; doi:10.1096/fba.2018-00044)
Supplement: Supplementary file 8 [file FBA2-1-283-s008.pdf]

Identification of hepatic NPC1L1 as an NAFLD-risk factor evidenced by ezetimibe-mediated steatosis prevention and recovery

Toyoda Y., Takada T. *et al.*

Supplemental Data

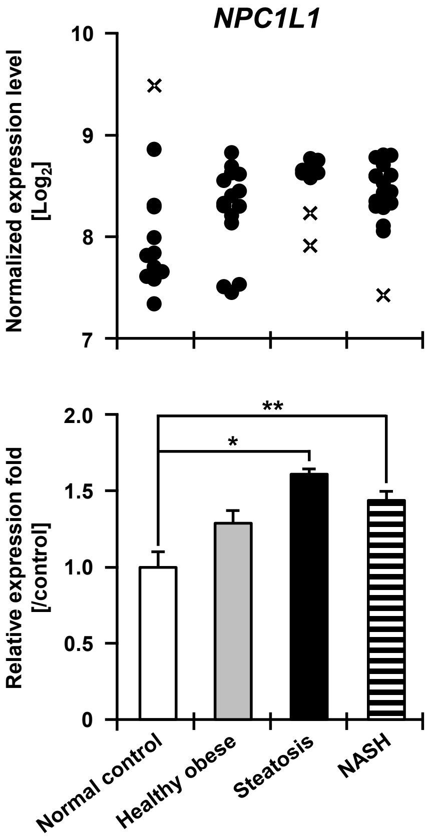

Fig. S8. NPC1L1 mRNA levels in the livers of healthy control subjects and patients with NAFLD.
To examine the NPC1L1-expression levels in the livers of patients with NAFLD, human hepatic transcriptome data from NAFLD patients and healthy controls that are available in the GEO database under accession number GSE48325 was accessed. Among the published data set, 19 samples for which the surgery status was "after surgery" were removed to minimize any unexpected effects of bariatric surgery operation on the hepatic gene profiling. In addition, a few outliers (cross marks in the *upper panel*) were removed (Smirnov–Grubbs test, $P < 0.05$). *Lower panel* shows NPC1L1 mRNA-expression levels in the livers of 11 Normal controls, 16 Healthy Obese subjects, 7 Steatosis patients, and 16 NASH patients as normalized fold changes. Data are expressed as the mean ± SEM. Statistical analyses for significant differences were performed using Bartlett's test, followed by a non-parametric Steel–Dwass test (*, $P < 0.05$; **, $P < 0.01$ among indicated groups).
